# Esophageal Obstruction from Food Bolus Impaction Successfully Managed with the “Upright Posture, Chin Tuck, Double Swallow” Maneuver: A Case Report

**DOI:** 10.5811/cpcem.19397

**Published:** 2024-07-28

**Authors:** Matthias Barden, Michael E. Schwartz

**Affiliations:** * Eisenhower Health, Emergency Medicine Residency Program, Racho Mirage, California; † Hi-Desert Medical Center, Department of Emergency Medicine, Joshua Tree, California; ‡ Desert Care Network, Section Gastroenterology, Coachella Valley, California

**Keywords:** gastrointestinal, esophageal obstruction, meat impaction, food bolus impaction

## Abstract

**Introduction:**

An attempt at medical management is often the initial step in addressing esophageal obstruction from an impacted food bolus. Medical management, however, has limited success and often requires urgent endoscopy. We present a case in which standard medical treatment failed, but a swallowing augmentation maneuver resolved the obstruction.

**Case Report:**

A 67-year-old female presented with esophageal obstruction after eating steak. Transfer to higher level of care for endoscopy was initiated; however, the receiving gastroenterologist suggested an “upright posture, chin tuck, double swallow” maneuver. This immediately resolved the patient’s symptoms, and she was discharged home.

**Conclusion:**

This case suggests a novel, non-endoscopic technique for esophageal obstruction from food bolus impaction.

## INTRODUCTION

Esophageal obstruction from impacted food bolus can cause significant discomfort, prevent oral intake, and be associated with serious complications.^1^ The failure of medical management may use significant resources, including transfer to an endoscopy-capable institution.^2^ Thus, many medical treatments have been proposed as ways to avoid the need for urgent endoscopy. In this case, some previously reported swallowing augmentation techniques generally used in dysphagia patients were combined to successfully resolve the obstruction and avoid a transfer for urgent endoscopy.

## CASE REPORT

A 67-year-old female presented to our emergency department with the complaint of esophageal obstruction. She denied any prior episodes but did report a history of known hiatal hernia. She was not taking any medications routinely and denied any past surgical history. The night prior to her presentation, she had been eating steak when she felt a bolus of meat become lodged in her upper esophagus. She made several attempts at swallowing and felt the bolus move lower in her chest but remain impacted there. Afterward she had odynophagia with any attempt to drink and experienced intermittent episodes of regurgitation. Her examination was benign, with stable vital signs and a soft, non-tender, non-distended abdomen.

Our facility lacks gastroenterology specialists on staff, and on the day of presentation general surgery was not available. We attempted medical management with normal saline 1 liter intravenous (IV), ondansetron 4 milligrams (mg) IV, and glucagon 2 mg IV. Thirty minutes after receiving glucagon, the patient was provided a carbonated beverage to drink. This worsened her discomfort and was followed by immediate regurgitation, but the sensation of obstruction was unchanged.

We contacted our health system’s transfer center and were connected with the gastroenterologist on call at one of our partner hospitals about 60 miles away. He agreed to receive the patient but suggested that prior to transfer a specific series of swallowing augmentation maneuvers be attempted. He suggested that the patient perform an “upright posture, chin tuck, double swallow” with water. The patient was provided a glass of water, instructed to stand in a straight upright position, flex her neck to tuck her chin to her chest, swallow some of the water, and then once it started to go down, initiate a second effort of swallowing.

This technique resulted in immediate and complete resolution of the patient’s symptoms. Afterward, she tolerated intake by mouth without difficulty. She was discharged home with a prescription for 40 mg pantoprazole daily and was given instructions for out-patient gastroenterology follow-up. Further information about her subsequent course was unavailable.

## DISCUSSION

Many methods have been proposed to avoid resorting to urgent endoscopy with esophageal obstruction from impacted food bolus. There is a low rate of success reported for the commonly attempted options, such as benzodiazepines, proteolytic enzymes, glucagon,^3^ nitroglycerin,^4^ and carbonated beverages.^5^ All these methods rely on the patient ultimately being able to complete the act of swallowing to relieve the obstruction. There are numerous techniques described to augment the process of swallowing for patients with neurologic, anatomic, or post-surgical causes of dysphagia.^6^ However, similar techniques have not previously been reported for esophageal obstruction from food bolus impactions.

Some of the specific techniques that have been described include upright posture,^7^ chin tuck position,^8^ and “double swallowing”.^9^ The consulting gastroenterologist on this case suggested that all three of those modalities be combined at the same time. While pretreatment with glucagon and providing a carbonated beverage had failed, the addition of instructing the patient on this “upright posture, chin tuck, double swallow” technique succeeded in relieving the obstruction, avoiding the need for transfer for urgent endoscopy.

## CONCLUSION

This case suggests the “upright posture, chin tuck, double swallow” technique may benefit patients with esophageal meat impaction and potentially avoid urgent endoscopy. Some of the usual medical interventions were attempted in this case and were unsuccessful in resolving the obstruction. This is only one case of a successful, augmented swallowing maneuver to relieve an esophageal obstruction, but the technique is easy to perform and, if successful, can avoid the need for urgent endoscopy.

**CPC-EM Capsule**

What do we already know about this clinical entity?

*There is a low rate of success for medical management of esophageal meat impaction. Failed medical treatment requires urgent endoscopy.*

What makes this presentation of disease reportable?

*A novel application of dysphagia treatment strategies was used successfully for esophageal meat impaction after standard medical management had failed.*

What is the major learning point?

*Dysphagia patients benefit from instruction on positioning and swallowing techniques. Those same instructions may help patients with esophageal meat impaction.*

How might this improve emergency medicine practice?

*Instruction on the “upright posture, chin tuck, double swallow” technique is simple and could potentially avoid the need for urgent endoscopy*
